# Enhancing Diagnostic Precision: Evaluation of Preprocessing Filters in Simple Diffusion Kurtosis Imaging for Head and Neck Tumors

**DOI:** 10.3390/jcm13061783

**Published:** 2024-03-20

**Authors:** Yuki Nakamitsu, Masahiro Kuroda, Yudai Shimizu, Kazuhiro Kuroda, Yuuki Yoshimura, Suzuka Yoshida, Yoshihide Nakamura, Yuka Fukumura, Ryo Kamizaki, Wlla E. Al-Hammad, Masataka Oita, Yoshinori Tanabe, Kohei Sugimoto, Irfan Sugianto, Majd Barham, Nouha Tekiki, Junichi Asaumi

**Affiliations:** 1 Radiological Technology, Graduate School of Health Sciences, Okayama University, Okayama 700-8558, Japan; pipj9cdx@s.okayama-u.ac.jp (Y.N.); pu7n0g7a@s.okayama-u.ac.jp (Y.Y.); tanabey@okayama-u.ac.jp (Y.T.);; 2 Department of Oral and Maxillofacial Radiology, Graduate School of Medicine, Dentistry, and Pharmaceutical Sciences, Okayama University, Okayama 700-8558, Japanasaumi@md.okayama-u.ac.jp (J.A.); 3 Department of Health and Welfare Science, Graduate School of Health and Welfare Science, Okayama Prefectural University, Okayama 719-1197, Japan; 4 Radiology Diagnosis, Okayama Saiseikai General Hospital, Okayama 700-8558, Japan; 5 Department of Radiology, Matsuyama Red Cross Hospital, Matsuyama 790-8524, Japan; 6 Department of Oral Medicine and Oral Surgery, Faculty of Dentistry, Jordan University of Science and Technology, Irbid 22110, Jordan; 7 Graduate School of Interdisciplinary Sciences and Engineering in Health Systems, Okayama University, Okayama 770-8558, Japan; oita-m@cc.okayama-u.ac.jp; 8 Department of Oral Radiology, Faculty of Dentistry, Hasanuddin University, Sulawesi 90245, Indonesia; irfansugianto@unhas.ac.id; 9 Department of Dentistry and Dental Surgery, College of Medicine and Health Sciences, An-Najah National University, Nablus 44839, Palestine; majd.barham@najah.edu

**Keywords:** diffusion-weighted image, Gaussian filter, head and neck tumor, magnetic resonance imaging, mean kurtosis, median filter, nonlocal mean filter, phantom, simple diffusion kurtosis imaging, restricted diffusion-weighted image

## Abstract

**Background:** Our initial clinical study using simple diffusion kurtosis imaging (SDI), which simultaneously produces a diffusion kurtosis image (DKI) and an apparent diffusion coefficient map, confirmed the usefulness of SDI for tumor diagnosis. However, the obtained DKI had noticeable variability in the mean kurtosis (MK) values, which is inherent to SDI. We aimed to improve this variability in SDI by preprocessing with three different filters (Gaussian [G], median [M], and nonlocal mean) of the diffusion-weighted images used for SDI. **Methods:** The usefulness of filter parameters for diagnosis was examined in basic and clinical studies involving 13 patients with head and neck tumors. **Results:** The filter parameters, which did not change the median MK value, but reduced the variability and significantly homogenized the MK values in tumor and normal tissues in both basic and clinical studies, were identified. In the receiver operating characteristic curve analysis for distinguishing tumors from normal tissues using MK values, the area under curve values significantly improved from 0.627 without filters to 0.641 with G (σ = 0.5) and 0.638 with M (radius = 0.5). **Conclusions:** Thus, image pretreatment with G and M for SDI was shown to be useful for improving tumor diagnosis in clinical practice.

## 1. Introduction

For the diagnosis of cerebral infarction and tumors using magnetic resonance imaging (MRI), the usefulness of the apparent diffusion coefficient (ADC) map in diffusion-weighted imaging (DWI) has been well established in routine clinical practice [1,2,3,4]. Recently, diffusion kurtosis imaging (DKI), a type of restricted diffusion-weighted (RD) imaging, has garnered attention, and its usefulness in the clinical image-based diagnosis of neurodegenerative diseases, ischemic stroke, and tumors has been reported [5,6,7,8,9,10,11,12]. Recently, the combination of intravoxel incoherent motion (IVIM) and amide proton transfer-weighted imaging in addition to DKI has been shown to improve the diagnostic performance by predicting lymph node metastasis in cervical cancer [13], in distinguishing among histological grades and clinical stages in clear cell renal cell carcinoma [14], and in predicting molecular subtypes in breast cancer [15,16].

DKI requires DWI with three or more different b-values in 30 axial directions at each b-value, which significantly increases the imaging time compared with that of ADC mapping. In addition, dedicated software, such as MATLAB, is required for its image analysis. For these reasons, DKI has not yet been applied in routine clinical practice.

To resolve these problems and to promote the use of DKI in daily clinical practice, we developed simple diffusion kurtosis imaging (SDI) [17,18,19], which produces a diffusion kurtosis (DK) image simultaneously with the ADC map. In SDI, a DK image is created using diffusion-weighted (DW) images with three b-values in three axial directions. They are currently used for the short-time imaging of ADC maps in daily clinical practice and involve analyses that implement general-purpose inexpensive software, such as Excel (2019; Microsoft Corp, Redmond, WA, USA) and ImageJ (1.51h; National Institutes of Health, Bethesda, MD, USA). SDI has been reported to be useful for the diagnosis of head and neck malignancies [19] and cystic diseases [20] in clinical studies, whereas DK images created from DW images, which have a low signal content, have been reported to vary in mean kurtosis (MK) values, which is inherent to SDI [19,20].

Recently, the usefulness of preprocessing with a Gaussian filter (G) [21,22], median filter (M) [23], and nonlocal mean filter (N) [24,25] for magnetic resonance (MR)-based diagnosis [21,22,23] and noise reduction [24,25] in MRI has been reported.

The present study investigated the effect of preprocessing using these three types of filters on removing the variability in MK values from DK images in SDI, using a standard phantom [26,27] for RD imaging, and sought to clarify the optimal setting values of the filter parameters. Furthermore, in clinical studies on SDI, we investigated whether filtering with these filter parameters improved the diagnostic ability for head and neck malignancies, by investigating whether the area under the curve (AUC) value, which indicates the ability to discriminate between tumors and normal tissues, was improved.

## 2. Materials and Methods

### 2.1. Phantom

A polyethylene glycol (PEG) phantom, which is a standard phantom for RD imaging, was developed by Khasawneh et al. [26]. The phantom can reproduce a wide range of MK values from normal to tumor tissues in clinical DK images at different PEG concentrations [26]. The phantom consisted of PEG (cat. no. P3640-500G; Sigma-Aldrich; Merck KGaA, Darmstadt, Germany), 0.03% *w*/*w* NaN_3_ as a preservative, and saline. Phantoms of 0, 40, 80, and 120 mM PEG concentrations were used: 0 and 40 mM, and 80 and 120 mM phantoms were used to represent normal and tumor tissues, respectively.

### 2.2. Patients

The study included 27 patients (12 men and 15 women; age range of 17–92 years, mean of 68 years) who underwent head and neck MRI examination as part of routine clinical practice for suspected head and neck mass lesions between March 2019 and September 2021 and who were diagnosed with tumor lesions based on pathology. The exclusion criteria included cases with a diameter of 10 mm or less (10 cases), cases with a strong artifacts on imaging of the tumor region (3 cases), and a case with metastatic cancer (1 case).

The study was approved by the Ethics Committee of the Okayama University Graduate School of Medicine, Dentistry, and Pharmaceutical Sciences and the Okayama University Hospital (protocol code Lab 2011-041). MRI was performed with the written informed consent of the patients.

### 2.3. MRI Devices and Sequences

#### 2.3.1. Phantom Imaging Conditions

A 3.0 T MRI device (MAGNETOM Prisma VE11C; Siemens Healthcare, Munich, Germany) with a 20-channel head/neck coil was used. DWI was performed using the RESOLVE sequence. The representative parameters were as follows: repetition time/echo time, 8000/91 ms; slice thickness, 5 mm; field of view, 120 × 120 mm; matrix, 224 × 224; bandwidth, 399 Hz/pixel; diffusion mode, three scan traces; readout segments, seven; b-value, 0, 400, and 800 s/mm^2^; and directions, 3.

The temperature of the phantom was adjusted to ca. 37 °C, similar to that in the human body, using a phantom-heating device [26,27] and an optical fiber thermometer (Fluoroptic™ m3300; LumaSense Technologies Inc., Milpitas, CA, USA), which was installed in the phantom for real-time phantom temperature measurements during MRI.

#### 2.3.2. Clinical Imaging Conditions

The MRI devices used for the imaging of patients were a 3 T MAGNETOM Skyra, 3 T MAGNETOM Prisma, 3 T MAGNETOM Verio, and 1.5 T MAGNETOM Aera (Siemens Healthcare). MRI was performed using head and neck coils. Axial DWI was performed using the RESOLVE sequence with short tau inversion recovery (STIR) for fat suppression. The representative parameters were as follows: repetition time/echo time, 6990–12,300/55–84 ms; slice thickness, 3 mm; gap, 4 mm; field of view, 200 × 200 mm; matrix, 140 × 140, 128 × 128, and 126 × 126; bandwidth, 990 Hz/pixel; diffusion mode, 3 scan traces; readout segments, 3; b-value, 0, 400, and 800 s/mm^2^; and directions, 3. The average DWI time was 390 (205–769) s. The generalized auto-calibrating partially parallel acquisition (GRAPPA) was set to 2.0, as the parallel imaging reduction factor. In addition to DW images, T1-weighted, contrast-enhanced T1-weighted, T2-weighted, and STIR MR images were obtained as part of the routine clinical practice.

### 2.4. Creation of Phantom DW Images

Twelve DW images were obtained with three different b-values (0, 400, and 800 s/mm^2^) and four different PEG phantom concentrations (0, 40, 80, and 120 mM). For each of the four phantoms, a 16 × 16 pixel region-of-interest (ROI) section was cropped.

**Figure 1:**
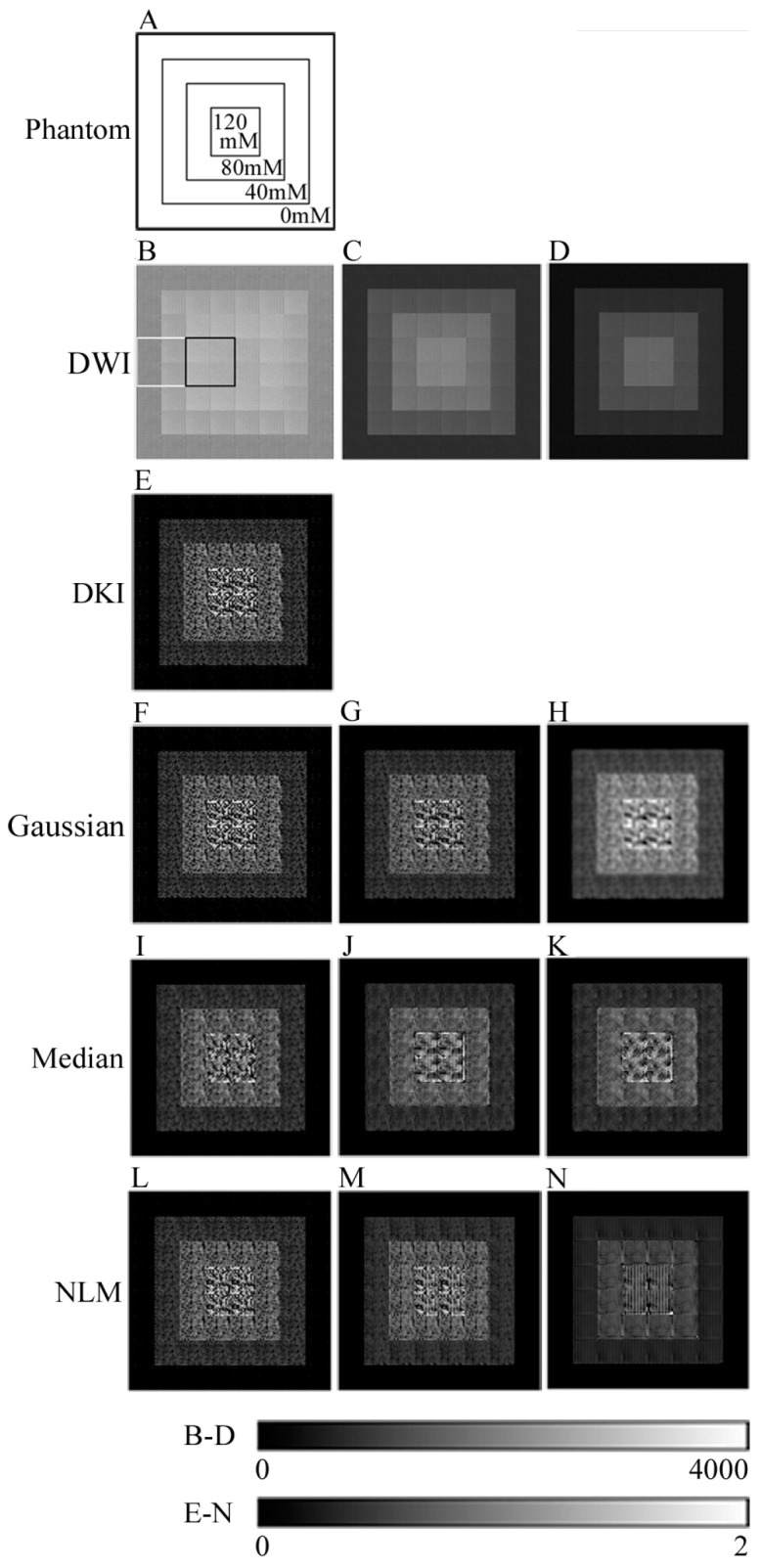
Diffusion-weighted (DW) images, diffusion kurtosis (DK) images, and pre-filter processing DK images of the phantom. (**A**) Structure of the phantom. Each concentration represents the concentration of the polyethylene glycol phantom. (**B**) DW image (b-value = 0 s/mm^2^). The black line is the tumor region of interest (ROI); the white line is a normal tissue ROI. (**C**) DW image (b-value = 400 s/mm^2^). (**D**) DWI (b-value = 800 s/mm^2^). (**E**) DK image. (**F**) Pre-Gaussian filter processing DK image (σ = 0.1). (**G**) Pre-Gaussian filter processing DK image (σ = 0.5). (**H**) Pre-Gaussian filter processing DK image (σ = 1.0). (**I**) Pre-median filter processing DK image (radius = 0.5). (**J**) Pre-median filter processing DK image (radius = 1.0). (**K**) Pre-median filter processing DK image (radius = 1.5). (**L**) Pre-nonlocal mean filter (NLM) processing DK image (σ = 5). (**M**) Pre-NLM processing DK image (σ = 15). (**N**) Pre-NLM processing DK image (σ = 30).

As shown in Figure 1A, multiple PEG density ROIs were combined to create a 128 × 128 pixel phantom DW image with eight ROIs in the vertical and horizontal directions. Figure 1B–D show the phantom DW images with the b-values of 0, 400, and 800 s/mm^2^. Phantom DK images and ADC maps were created based on the DW images.

### 2.5. Preprocessing of DW Images with Filters and Parameter Setting

In this study, the effects of three types of image-smoothing filters, namely G, M, and N, were examined. Pre-filter processing DK images were created by applying G, M, and N preprocessing to each of the three DW images (with b-values 0, 400, and 800 s/mm^2^) used to create the DK images.

G (URL: https://imagej.nih.gov/ij/docs/guide/146-29.html#toc-Subsection-29.11, accessed on 24 January 2024) was used to smooth the image as follows. For the target pixel, the filter weight coefficients were set to approximate a Gaussian distribution to assign a higher weight to the center, as expressed in Equation (1) [28].

g(x, y) = e^[−(x^2^ + y^2^)/2σ^2^] (1)

where g is the filter weight coefficient, and x and y are the spread of pixels. The filter parameter “σ” is the standard deviation, with which the degree of smoothing varies.

M (URL: https://imagej.nih.gov/ij/docs/guide/146-29.html#toc-Subsection-29.11, accessed on 24 January 2024) smooths the image by replacing the value of the target pixel with the median value of a group of pixels near the target pixel. The filter parameter “radius” indicates the range of the area over which the median is calculated.

N [22] is a filter that smooths the image by collecting the center pixels of the target block centered on the pixel-of-interest and the reference block with high similarity, assigning them a large weight if the similarity between the blocks is high and a small weight if it is low, which is used to calculate the weighted average of the center pixels of the surrounding blocks, as expressed in Equation (2) [29].

n(x, y) = e^[−max (d^2^ − 2σ^2^, 0.0)/h^2^] (2)

where n is the filter weight coefficient, x is the pixel of interest, y is any pixel in the search area, d is the Euclidean distance (distance between two pixels), and h is a filtering parameter set according to the value of “σ”. The filtering parameter “σ” is the standard deviation and the degree of smoothing varies with “σ”.

As preprocessing steps for DW images using G and M, the DW images were treated with the G and M filtering functions using ImageJ. For the preprocessing steps using N, the DW images were treated using the N plugin function in ImageJ developed by the Biomed group (URL: https://sites.imagej.net/Biomedgroup/plugins, accessed on 24 January 2024).

In the phantom study of the filtering parameters, G was set to σ = 0.1–1.0, with a total of 10 steps at 0.1 intervals; M was set to a radius = 0.5–1.5 in three steps at 0.5 intervals, and N was set to σ = 1, 2, 3, 4, 5, 6, 9, 10, 15, 20, 25, and 30, in 12 steps. These specific filter parameters and intervals were selected in a preliminary study. Based on the results of the phantom study, G was set to σ = 0.5, M was set to a radius = 0.5, and N was set to σ = 15 for the clinical study.

### 2.6. DK Image Creation

In this paper, DK images were created by the SDI method [17,18,19,20] using the DW images of phantoms and clinical cases, which were obtained for ADC map creation during routine clinical practice. A DK image was defined as an image for which the MK values were calculated using the DW images at three b-values (0, 400, and 800 s/mm^2^) using the SDI method. In SDI, DW images with multiple b-values can be used for the simultaneous creation of a DK image and an ADC map. The SDI software (v1.0) included ImageJ (1.51h; NIH) and Microsoft Excel (2019; Microsoft).

For each pixel in the DW image at the three different b-values (0, 400, and 800 s/mm^2^), each signal value was logarithmically converted and plotted on the vertical *Y*-axis. The b- values were plotted on the horizontal *X*-axis. These values were approximated using the quadratic function y = Ax^2^ + Bx + C, and the quadratic and linear coefficients A and B, respectively, were calculated. Equation (3) was used to obtain the MK value for each pixel, which was then used to create a DK image using the ImageJ software.

MK = 6 × A/(−B)^2^ (3)

### 2.7. Setting the ROI for the Evaluation

#### 2.7.1. Setting the ROI of the Phantom Image

As shown in Figure 1B, 32 × 32 pixel ROIs were set in the PEG phantom of 80 and 120 mM as the tumor ROI and the PEG phantom of 0 and 40 mM as the normal ROI in the phantom DK image for evaluation.

#### 2.7.2. Setting the ROI for the Clinical Study

For each case, a tumor ROI was set in the tumor area using a slice section with the largest tumor area and a DW image with a b-value of 0 s/mm^2^. A normal ROI was set in the masseter muscle, which was clearly delineated in the slice with the largest tumor area. If the masseter muscle was unclear, indistinct, or out of imaging range, the most clearly delineated muscles among the temporalis, erector spinae, or lateral pterygoid muscles were used. These ROIs were set by the consensus of three radiologists (M.K., J.A., and Y.S., with 39, 26, and 6 years of experience in diagnostic imaging, respectively). STIR and T2-weighted images were used as references when necessary. These ROIs were used to evaluate the DK images.

### 2.8. Image Analysis

MK values within the ROI of the DK images were compared with and without each filter.

The Kolmogorov–Smirnov test was used to examine the normality of the MK values in the ROIs. For comparisons of MK values among ROIs, the Kruskal–Wallis test and Holm’s multiple comparisons were used. The Fligner–Killeen homogeneity of variance test was used to compare the equal variances of the MK values among the ROIs. For the assessment of discriminability between the tumor and normal ROIs, a receiver operating characteristic (ROC) curve analysis was used, and the AUC was examined. The significance of differences between ROC curves for the presence and absence of each filter was assessed. AUC values of 1.0–0.9, 0.9–0.8, 0.8–0.7, 0.7–0.6, and 0.6–0.5 were evaluated as “excellent”, “very good”, “good”, “satisfactory”, and “unsatisfactory”, respectively.

### 2.9. Statistical Analysis

R (v4.2.3, URL: https://www.r-project.org/, accessed on 24 January 2024) was used for the permutation test, Levene’s test, Fligner–Killeen variance homogeneity test, and ROC analyses. EZR (v1.61, URL: https://www.jichi.ac.jp/saitama-sct/SaitamaHP.files/download.html, accessed on 1 April 2023.) was used for the Kruskal–Wallis test, Holm’s multiple comparisons, Kolmogorov–Smirnov test, and the comparison of ROC curves. A *p*-value < 0.05 was considered a statistically significant difference.

## 3. Results

### 3.1. Changes in Median MK Values in the Phantoms

Figure 1E shows a phantom DK image obtained via the SDI method, using the phantom DW images shown in Figure 1B–D. Figure 1F–H show the DK images obtained using the preprocessed DW images of Figure 1B–D with G with σ = 0.1, 0.5, and 1.0, respectively. Panels I, J, and K in Figure 1 are the DK images created using the preprocessed DW images of Figure 1B–D obtained with M of radius = 0.5, 1.0, and 1.5, respectively. Panels L, M, and N in Figure 1 are the DK images created using the preprocessed DW images of Figure 1B–D obtained with N of σ = 5, 15, and 30, respectively. Increasing σ or the radius smoothed the DK image.

Table 1 lists the changes in the median MK values of the tumor and normal ROIs when the parameters of the three types of filters in the phantom were varied. We used a Kruskal–Wallis test with a Holm’s test to compare the median MK values of the tumor and normal ROIs in images obtained with and without a filter. For the range of σ and radius values used, no significant change in the MK values for either the tumor or normal ROIs, for any of the filters, were observed.

### 3.2. Changes in Variance and AUC in the Phantoms

Table 2 shows the variance in MK values in the tumor and normal ROIs, and the AUC values of the ROC analysis to describe the discriminability between the tumor and normal tissues when the parameters of G, M, and N were varied in the phantom.

We used the Fligner–Killeen homogeneity of variance test to compare the variance in MK values in the tumor and normal ROIs between the images obtained with and without a filter. Increases in σ and the radius decreased the variance in MK values in the tumor ROI.

We used the AUC values in the ROC curve analysis to assess the discriminability between the tumor and normal ROIs. Increases in σ and the radius increased the AUC values, indicating an increase in the ability to discern between the tumor and normal tissues.

The variance in the MK value of the tumor ROI was significantly reduced for σ ≥ 0.4 for G, radius ≥ 0.5 for M, and σ ≥ 2 for N. The AUC values increased significantly for σ ≥ 0.3 for G, radius ≥ 0.5 for M, and σ ≥ 2 for N. The AUC values were “excellent” at ≥0.9 for σ ≥ 0.5 for G, σ ≥ 0.5 for M, and σ ≥ 15 for N.

Based on these results, the following parameter values, which indicated “excellent” AUC values, were used in the clinical analysis: σ = 0.5 for G, radius = 0.5 for M, and σ = 15 for N.

### 3.3. Clinical Case Information

Table 3 shows information on the clinical cases and the ROI setup. Thirteen malignant tumors were included in the study after the exclusion criteria were applied; they included eight squamous cell carcinomas (SCCs), two adenoid cystic carcinomas, one acinic cell carcinoma, one malignant lymphoma, and one osteosarcoma.

The total number of pixels in the ROI set up for each case was 2702 (mean: 208; median: 219) for the tumor ROIs and 2142 (mean: 165; median: 177) for the normal ROIs. A permutation test (*p* = 0.3282) and Levene’s test (*p* = 0.1525) were used to test for equal medians and variances. According to these results, the respective MK values of all pixels of the tumor ROI and all pixels of the normal ROI in each case were pooled and used for the subsequent analysis of the MK values (the so-called “pixel analysis”).

**Figure 2:**
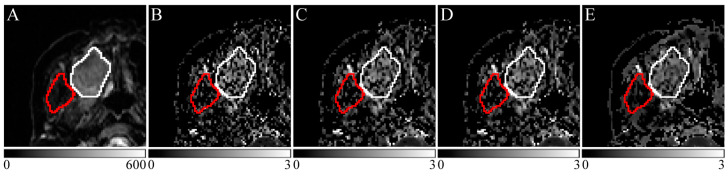
Clinical images of case 9. (**A**) Diffusion-weighted (DW) image (b = 0 s/mm^2^). (**B**) Diffusion kurtosis (DK) image. (**C**) Pre-Gaussian filter processing DK image (σ = 0.5). (**D**) Pre-median filter processing DK image (σ = 0.5). (**E**) Pre-nonlocal mean filter (NLM) processing DK image (σ = 15). The white line indicates the tumor region of interest (ROI), and the red line indicates a normal ROI surrounding the right temporalis muscle.

In each case, DK images were created from the DW images with b-values of 0, 400, and 800 s/mm^2^. Images of case 9 are shown in Figure 2. The DK images in each ROI were smoothed by preprocessing the DW images with G with σ = 0.5, M with radius = 0.5, and N with σ = 15, which had yielded “excellent” AUC values in the phantom study.

### 3.4. MK Values for Tumor and Normal ROIs in Clinical Practice

**Figure 3:**
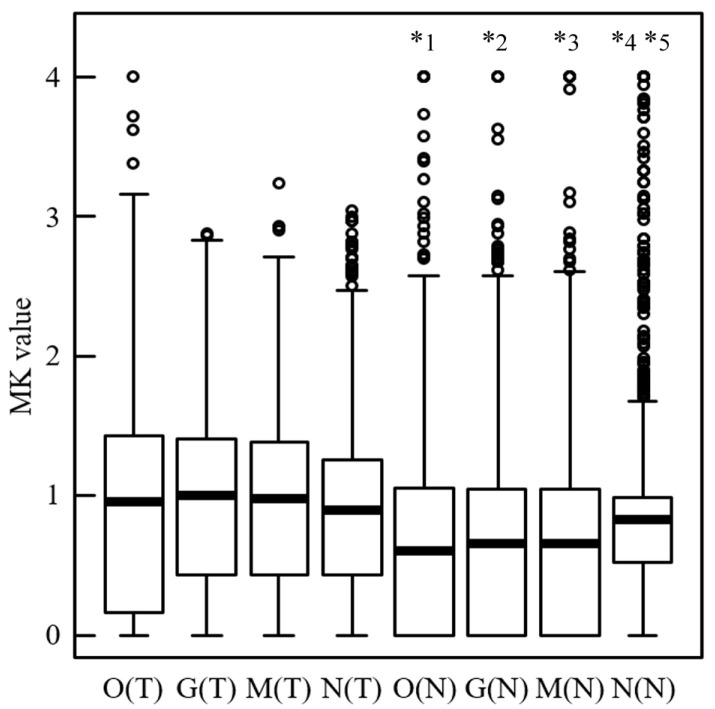
Mean kurtosis (MK) values of the tumor and normal regions of interest (ROIs) in clinical practice. Box-and-whisker diagram of the MK values of the tumor and normal ROIs in the pixel analysis. The vertical axis is the MK value. The horizontal thick line in each box represents the median (50th percentile) of the measured values; the top and bottom of the box represent the 25th and 75th percentiles, respectively; and the whiskers indicate the range of observed data points from the maximum to minimum within the 1.5 quartile range indicated by the box. Circles indicate outliers. O(T): unfiltered diffusion kurtosis (DK) image for the tumor tissue; G(T): pre-Gaussian filter processing DK image for the tumor tissue; M(T): pre-median filter processing DK image for the tumor tissue; N(T): pre-nonlocal mean filter (NLM) for the tumor tissue processing DK image; O(N): unfiltered DK image for the normal tissue; G(N): pre-Gaussian filter processing DK image for the normal tissue; M(N): pre-median filter processing DK image for the normal tissue; N(N): pre-NLM processing DK image for the normal tissue. *p*-values reflect comparisons between two groups using the Kruskal–Wallis test and Holm‘s method. *1 for O(T), *2 for G(T), *3 for M(T), *4 for N(T), and *5 for O(N), indicating a significant difference of *p* < 0.001.

Figure 3 shows the MK values for the tumor and normal ROIs in the pixel analysis. The MK values of the tumor ROIs were not significantly different according to whether any type of filter had been used or not. The MK values of the normal ROIs were significantly different (*p* < 0.05) between those with and without the N filter only. No significant differences between the groups with and without G or M filters were observed. A significant difference was noted between the tumor and normal ROIs, regardless of the filter.

The variance in MK values between the images obtained with and without each filter was improved (*p* < 0.001) for every filter according to the Fligner–Killeen test for the tumor and normal ROIs, respectively.

### 3.5. Distinguishability between Tumor and Normal Tissues in Clinical Practice

**Figure 4:**
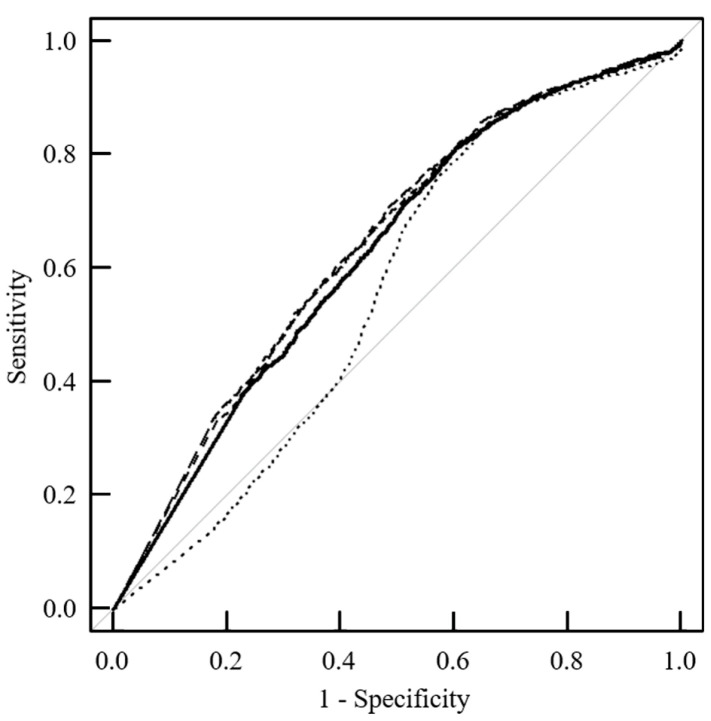
Distinguishability between the tumor and normal tissues in clinical practice. Receiver operating characteristic (ROC) curves based on the pixel analysis of the mean kurtosis (MK) values for the tumor and normal ROIs are indicated as a solid line (without filter), long dashed line (Gaussian filter), dashed line (median filter), and dotted line (nonlocal mean filter).

Figure 4 shows the results of the ROC analysis of the distinguishing power between the tumor and normal tissues. The AUC values of 0.641 (*p* < 0.001) for the implementation of G and 0.638 (*p* < 0.05) for the implementation of M filters significantly improved the distinguishing power, compared to that of 0.627 without filters. In contrast, the implementation of the N filter significantly worsened the AUC to 0.558 (*p* < 0.001).

## 4. Discussion

This report revealed the clinical utility of using preprocessing filters in DKI based on SDI. In a basic study using phantoms, DW images were preprocessed using G, M, and N filters to determine the optimal filter parameters that could reduce the variation in the MK values in DK images. In our clinical study, the pretreatment of DW images with the G or M filters using the identified optimal parameters resulted in the homogenization of the MK values of the tumor and normal tissues, without significant changes in the median MK values, and resulted in a significantly improved discrimination between the tumor and normal tissues.

Recently, SDI has been reported as a method for obtaining DK images [18,19,20]. In a study on SDI, Hamada et al. [17] reported a method for creating DK images using two common and inexpensive software packages: ImageJ and Excel. Kuroda et al. [18] reported a fast DKI method for simultaneously obtaining ADC maps and DK images of phantoms and healthy volunteers by DWI in three axes, with three b-values, using the low maximum b-value used for ADC map creation, in routine clinical practice, thereby reducing the imaging time. Using this SDI approach, Shimizu et al. [19] reported the usefulness of DKI in differentiating head and neck tumors from normal tissue. Fukumura et al. [20] reported the usefulness of DKI for differentiating cystic diseases of the head and neck. Previous reports on SDI pointed out that MK images have a noticeable variability in MK values, which is inherent to SDI [18,19,20]. The present study demonstrated the usefulness of using preprocessing filters in SDI, by revealing the homogenization of MK values and improving diagnostic performance in clinical practice.

For the basic study, we used a specially developed restricted diffusion standard PEG phantom [26,27]. The MK values of this phantom are similar to those of various tumors in previous clinical studies as noted with 3 T MRI, for example, 0.41 ± 0.09 for renal cancer [30], 0.50 ± 0.08 for grade II glioma [31], approximately 0.60 for brain tumors [32], approximately 0.58–0.67 for gastric cancer [33], 0.75 ± 0.22 for hepatocellular carcinoma [34], 1.00 ± 0.11 for rectal cancer [35], 0.92 ± 0.14 for squamous cell carcinoma, and 1.21 ± 0.26 for olfactory neuroblastoma [36], which reportedly covers a majority of tumor cases [26]. In the present study, a combination of two different concentrations of PEG phantoms was used when attempting to reproduce the heterogeneous clinical MK values in tumors and normal tissues. The MK value of the combination of PEG 80 mM and 120 mM phantoms for the tumor phantom in this study was 0.72, which was similar to that for brain tumors [32], gastric cancer [33], and hepatocellular carcinoma [34].

Variations in the MK values were observed for DK images with a small number of axes, such as SDI. Previously, several studies of DKI using filter processing have been reported [22,24,25,37]. The usefulness of implementing preprocessing filters for obtaining DW images, which are the source of DK images [22,24,37], and that of postprocessing filters for the created DK images have previously been reported [25]. To the best of our knowledge, no clinical study has compared the usefulness of processing with various filters. We revealed that prefilter processing improved the clinical diagnostic performance of DKI based on SDI.

Regarding DKI using preprocessing with G [22,37], Falangola et al. [22] reported age-related MR diffusion pattern features in the prefrontal cortical microstructure of the normal brain. Cao et al. [37] reported that DKI using preprocessing with G was effective in predicting the microvascular invasion and histological grade of hepatocellular carcinoma. Zhang et al. [24] reported that DKI using preprocessing with N was useful for quantifying DKI in various anatomical regions of the human brain and spinal cord. Reports on postprocessing filters for DK images are scarce. Zhou et al. [25] reported that postprocessing with N can efficiently remove noise while preserving the fine structure and detail of the normal brain. These previous reports did not provide any description of filter parameters that could be compared to those in the present study.

To the best of our knowledge, the optimal parameter settings for each filter that can improve diagnostic performance haven not been reported to date. A standard phantom for DKI [26,27] is necessary for such basic studies. The present study identified the optimal parameters for improving the diagnostic performance with each filter using the developed phantom [26,27] and confirmed their utility in a clinical study.

SDI has the features of a low maximum b-value and low number of axes, reducing imaging time, in contrast to conventional DKI. The literature has summarized the technical aspects of the DWI method. SDI [18,19,20] uses DW images captured with a maximum b-value of 800 s/mm^2^ in three axial directions for short-term imaging. DWI is widely used in daily clinical practice for producing ADC maps. The impact of a lower number of axes and a lower maximum b-value, compared to conventional DKI, has been validated in previous reports on SDI [18,19,20] based on a detailed literature review. Several studies have demonstrated the clinical utility of three-axis imaging for DKI [18,38,39], and Rosenkrantz et al. [39], in a review, summarized that three-axis imaging is sufficient for several imaging directions. As for imaging with a lower maximum b-value, several clinical studies have also shown the clinical utility of DKI with a lower maximum b-value [14,19,20,40,41] for clear cell renal cell carcinoma, head and neck cysts and tumors, rectal cancer, and prostate cancer. Clinical studies using a maximum b-value of 800 s/mm^2^ for SDI have also shown that DKI is useful for diagnosing head and neck tumors [19] and cystic diseases [20]. Setting a minimum b-value may affect the IVIM. If the consideration of the IVIM is required in DKI, it may be necessary to set the minimum b-value to a lower value, such as 100–200 s/mm^2^. However, most previous clinical studies have reported MK values with a minimum b-value of 0 s/mm^2^ for DK imaging [12]. In the present and previous studies of SDI [18,19,20], imaging with a minimum b-value of 0 s/mm^2^ was used to compare the MK values with those of previous reports.

One limitation of this study was the insufficient number of cases. In future, the number of cases should be increased to examine the accuracy of the results. The parameters used in the clinical study were optimized for basic research. Although the variance in MK values was improved mainly in tumors, a discrepancy was noted in the degree of improvement in the AUC values between the basic research and the clinical study. Further studies are needed to focus on the relationship between filter parameter optimization and AUC in clinical practice, based on a larger number of cases, which will improve the diagnostic performance of DK images. In addition, three types of filters were used in the study, all of which were used for preprocessing. Further research is required to improve the diagnostic performance of DK images, such as the use of other types of filters and postprocessing to investigate filters for DKI. Furthermore, the present study did not examine the impact of different ROI settings by multiple observers on the AUC; this might be an area for future work.

## 5. Conclusions

The basic and clinical investigations performed in this study showed that G and M preprocessing can improve the diagnostic performance of SDI, which is a type of DK imaging. This study demonstrated the utility of G and M preprocessing filters in SDI to improve the diagnostic performance of head and neck tumors, which has not been reported previously.

## Figures and Tables

**Table 1:** Variation in the median value of the phantom with filter parameters.

		Tumor ROI	*p*-Value	Normal ROI	*p*-Value
Filter Type	Parameter	Median (Q1, Q3)		Median (Q1, Q3)	
Gaussian	σ = 0	0.715 (0.327, 1.014)		0.055 (0.000, 0.346)	
	σ = 0.1	0.715 (0.327, 1.014)	1.00	0.055 (0.000, 0.346)	1.00
	σ = 0.2	0.715 (0.327, 1.014)	1.00	0.055 (0.000, 0.346)	1.00
	σ = 0.3	0.717 (0.334, 1.011)	1.00	0.058 (0.000, 0.347)	1.00
	σ = 0.4	0.710 (0.406, 0.981)	1.00	0.075 (0.000, 0.349)	1.00
	σ = 0.5	0.712 (0.504, 0.920)	1.00	0.104 (0.000, 0.358)	1.00
	σ = 0.6	0.718 (0.556, 0.879)	1.00	0.153 (0.000, 0.358)	1.00
	σ = 0.7	0.728 (0.597, 0.869)	1.00	0.179 (0.000, 0.356)	1.00
	σ = 0.8	0.731 (0.618, 0.856)	1.00	0.206 (0.000, 0.356)	1.00
	σ = 0.9	0.730 (0.632, 0.854)	0.76	0.225 (0.000, 0.353)	1.00
	σ = 1.0	0.733 (0.645, 0.845)	0.47	0.232 (0.000, 0.353)	1.00
Median	Radius = 0	0.715 (0.327, 1.014)		0.055 (0.000, 0.346)	
	Radius = 0.5	0.713 (0.525, 0.905)	1.00	0.027 (0.000, 0.351)	1.00
	Radius = 1.0	0.692 (0.562, 0.874)	1.00	0.066 (0.000, 0.352)	1.00
	Radius = 1.5	0.701 (0.558, 0.855)	1.00	0.066 (0.000, 0.346)	0.74
NLM	σ = 0	0.715 (0.327, 1.014)		0.055 (0.000, 0.346)	
	σ = 1	0.723 (0.317, 1.013)	1.00	0.051 (0.000, 0.344)	1.00
	σ = 2	0.723 (0.393, 0.982)	1.00	0.031 (0.000, 0.344)	1.00
	σ = 3	0.707 (0.430, 0.938)	1.00	0.041 (0.000, 0.351)	1.00
	σ = 4	0.705 (0.451, 0.951)	1.00	0.067 (0.000, 0.367)	1.00
	σ = 5	0.703 (0.458, 0.951)	1.00	0.061 (0.000, 0.354)	1.00
	σ = 6	0.714 (0.447, 0.952)	1.00	0.077 (0.000, 0.360)	1.00
	σ = 9	0.718 (0.457, 0.962)	1.00	0.049 (0.000, 0.358)	1.00
	σ = 10	0.718 (0.454, 0.946)	1.00	0.030 (0.000, 0.353)	1.00
	σ = 15	0.732 (0.514, 0.927)	1.00	0.024 (0.000, 0.351)	1.00
	σ = 20	0.741 (0.555, 0.904)	1.00	0.067 (0.000, 0.354)	1.00
	σ = 25	0.727 (0.578, 0.892)	1.00	0.092 (0.000, 0.364)	1.00
	σ = 30	0.718 (0.583, 0.893)	1.00	0.057 (0.000, 0.360)	1.00

ROI: region of interest; Q1: the lower quartile value under which 25% of data points are found, in increasing order; Q3: the upper quartile value under which 75% of data points are found, in increasing order; NLM: nonlocal means. *p* indicates the result of the Kruskal–Wallis test with Holm’s test to compare the signal values between the tumor and normal ROIs in the images obtained with and without a filter.

**Table 2:** Change in the variance in the mean kurtosis values and the area under the receiver operating characteristic curve values for each filter parameter in the phantom.

Filter		Homogeneity Evaluated by Fligner–Killeen Test		Discernment Ability
Filter Type	Filter Parameter	*p*-Value for Tumor ROI	*p*-Value for Normal ROI	AUC Value
Gaussian	σ = 0.0			0.835
	σ = 0.1	1.000	1.000	0.835
	σ = 0.2	0.999	1.000	0.835
	σ = 0.3	0.637	0.000	0.837 *
	σ = 0.4	0.000	0.000	0.865 *
	σ = 0.5	0.000	0.003	0.912 *
	σ = 0.6	0.000	0.567	0.948 *
	σ = 0.7	0.000	0.962	0.967 *
	σ = 0.8	0.000	0.502	0.978 *
	σ = 0.9	0.000	0.945	0.984 *
	σ = 1.0	0.000	0.854	0.988 *
Median	Radius = 0			0.835
	Radius = 0.5	0.000	0.000	0.919 *
	Radius = 1.0	0.000	0.000	0.956 *
	Radius = 1.5	0.000	0.001	0.965 *
NLM	σ = 0			0.835
	σ = 1	0.445	0.000	0.836
	σ = 2	0.041	0.000	0.858 *
	σ = 3	0.000	0.000	0.871 *
	σ = 4	0.000	0.000	0.881 *
	σ = 5	0.000	0.000	0.884 *
	σ = 6	0.000	0.000	0.880 *
	σ = 9	0.000	0.000	0.889 *
	σ = 10	0.000	0.000	0.896 *
	σ = 15	0.000	0.000	0.924 *
	σ = 20	0.000	0.000	0.957 *
	σ = 25	0.000	0.002	0.959 *
	σ = 30	0.000	0.000	0.965 *

ROI: region of interest; AUC: area under the receiver operating characteristic curve; NLM: nonlocal means. *p*-value indicates the results of the Fligner–Killeen homogeneity of variance test to compare the variance between the images obtained with and without a filter for the tumor and normal ROIs. The AUC value indicates the ability to discern between the tumor and normal tissues. * indicates a significant difference (*p* < 0.001) in AUC values between the tumor and normal ROIs in the images obtained with and without a filter.

**Table 3:** Case information and the site and number of pixels of the regions of interest.

		ROI Setting			
Case	Histological Classification	Tumor ROI *		Normal ROI **	
		Position	Number of Pixels	Position	Number of Pixels
1	Squamous cell carcinoma	Maxilla	434	Erector spinae muscle	40
2			334	Masseter muscle	119
3			219	Masseter muscle	214
4			132	Lateral pterygoid muscle	100
5		Mandible	63	Masseter muscle	87
6		Tongue	289	Masseter muscle	322
7			245	Masseter muscle	65
8			21	Erector spinae muscle	300
9	Adenoid cystic carcinoma	Palate	412	Temporal muscle	200
10			59	Masseter muscle	177
11	Acinic cell carcinoma	Parotid gland	223	Masseter muscle	110
12	Malignant lymphoma	Maxilla	154	Masseter muscle	186
13	Osteosarcoma	Mandible	117	Erector spinae muscle	222

ROI: region of interest. * ROI of the tumor tissue set on the slice, showing the maximum area of the tumor. ** ROI of the normal tissue set on the erector spinae, masseter, lateral pterygoid, or temporalis muscle on a slice of tumor ROI.

## Data Availability

The data presented in this study are available from the corresponding author upon reasonable request.
